# ﻿Typification of six names in *Camellia* (Theaceae)

**DOI:** 10.3897/phytokeys.201.84699

**Published:** 2022-06-16

**Authors:** Dongwei Zhao

**Affiliations:** 1 Department of Forestry, College of Forestry, Central South University of Forestry and Technology, 498 Shao-shan South Road, Changsha, Hunan 410004, China Central South University of Forestry and Technology Changsha China

**Keywords:** *Camellia*, lectotypification, *Thea*

## Abstract

Six names in *Camellia* or its synonym *Thea*, including Camelliareticulataf.simplex, *C.symplocifolia*, *Thea forrestii*, T.lanceolatavar.stenophylla, *T.megacarpa* and *T.yunnanensis* are lectotypified here with nomenclatural and taxonomic notes provided.

## ﻿Introduction

Species of *Camellia* L. (Theaceae) are of great economic value through the productions of tea and seed oil, as well as cultivation for their beautiful flowers. The genus has been taxonomically revised at least six times (e.g. global species: Sealy 1958; Chang 1981; Chang and Bartholomew 1984; Ming 2000; species in China: Chang 1998; Ming and Bartholomew 2007), but the types of many taxa remain unclear. Some of them have recently been typified (Zhao et al. 2017a, b, 2018, 2019; Zhao 2021, 2022). Following these works, six more names in the genus with similar problems are identified and they are lectotypified below.

## ﻿Materials and methods

Taxonomic literature, especially protologues, and specimens at Herbaria A, BM, E, G, K, KUN, L, MO, NY, P, PE, TCD, U and US (acronyms based on Thiers 2022, continuously updated) were examined. The most informative specimen was selected as the lectotype, based on Art. 9 of the Shenzhen Code (Turland et al. 2018, hereafter ICN).

## ﻿Typification of names

### ﻿1. Camellia reticulata Lindl. f. simplex

Taxon classificationPlantaeEricalesTheaceae

Sealy, Revis. Gen. Camellia 183. (1958)

26344B3D-859E-5B5C-9CAD-55EE811060B4

#### Lectotype.

**Figure 1.**
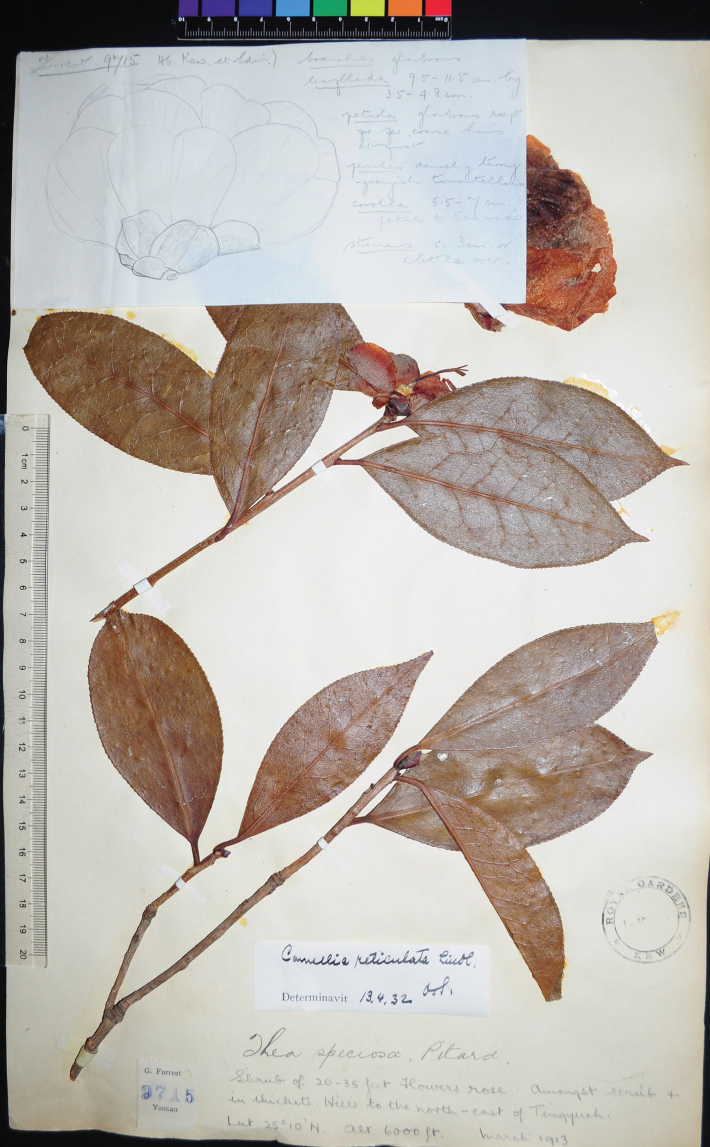
Lectotype of CamelliareticulataLindl.f.simplex Sealy (*G. Forrest 9715* at K). Photo: D.W. Zhao.

(designated here): China. Yunnan: Northeast of Tengyueh (Tengchong), 20°10'N, 6000 ft alt., March 1913, *G. Forrest 9715* (K!; Fig. 1).

#### Notes.

Sealy (1958: 183) established the new form to represent the wild plants of *C.reticulata* since the latter was described based on a plant with semi-double flowers (Zhao 2022). A gathering, *Forrest 9715*, was designated as the type of the form. Sealy (1958: 182) cited “*Forrest 9715* (E, K)” on the page before the protologue, so the collection *Forrest 9715* at Herbaria E and K are syntypes of C.reticulataf.simplex (Art. 40 Note 1 of the ICN). A single sheet of *Forrest 9715* was found at E with barcode E00117770 (see http://data.rbge.org.uk/herb/E00117770). However, the single specimen of the collection at K bears Sealy’s drawing of a flower and his notes on the morphology, so it is selected above as the lectotype of the form (Fig. 1).

Ming (2000: 278) recognised C.reticulataf.simplex as a heterotypic synonym of *C.reticulata*. Though the original material of the species was derived from the cultivated plant, the current broad circumscription for *C.reticulata* covers wild plants under the ICN since the nomenclatural type does not need to be the most typical or representative of a natural species (Art. 7.2 of the ICN). However, it is better to name cultivars according to the *International Code of Nomenclature for Cultivated Plants* (Art. 28 Note 2; Brickell et al. 2016).

### ﻿2. Camellia symplocifolia

Taxon classificationPlantaeEricalesTheaceae

Griff., Itin. Pl. Khasyah Mts. 40, No. 652. (1848)

70440A9D-96FF-5208-A2EE-139008B01E40

#### Lectotype.

**Figure 2.**
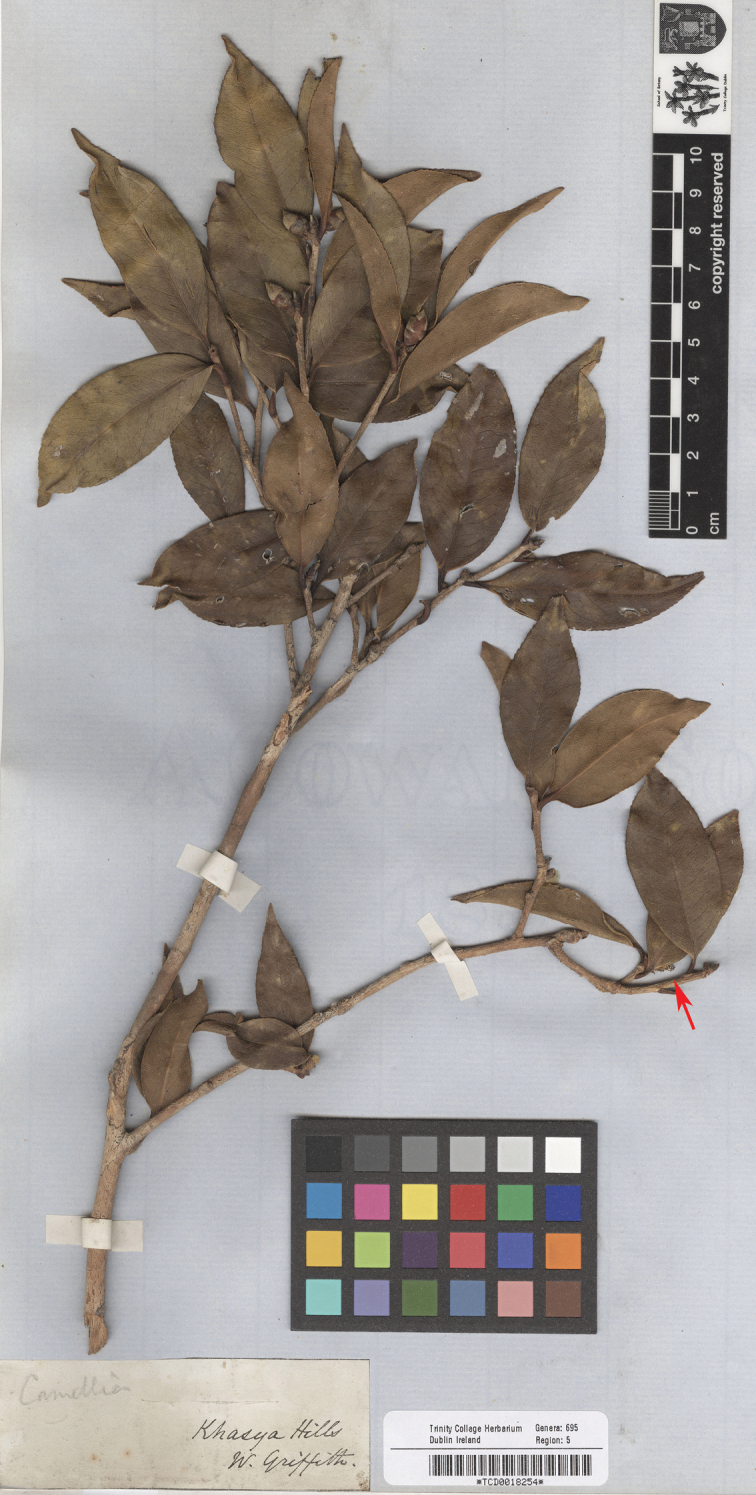
Lectotype of *Camelliasymplocifolia* Griff. (*Griffith s.n.*, TCD0018254). The red arrow indicates one position of the androecium and gynoecium. Image scanned by Ms J. Stone (TCD) and adapted by D.W. Zhao.

(designated here): India. Khasya Hills, *W. Griffith s.n.* (TCD0018254!; Fig. 2).

#### Notes.

Griffith (1848) described *C.symplocifolia* under his catalogue number 652 and stated that the original material was collected at Churra, Khasyah. Though “Icon no. 31” was cited in the protologue and this citation directs to a drawing, fig. 2 of plate DCIV (“*C.simplicifolia*”) in *Icones Plantarum Asiaticarum* (Griffith 1854), the single drawing cannot be considered as the holotype because the corresponding specimens that were collected and used by Griffith to prepare the description, although uncited, may still exist (see Art. 9 Ex. 2 of the ICN). Furthermore, fig. 2 of plate DCIV (Griffith 1854) is a poor drawing of leaves and flowers, which can hardly illustrate the key features of the taxon and is, therefore, unsuitable to serve as the lectotype. The specimens of potential original material collected by Griffith in Khasya Hills were found at BM, K and TCD. Unfortunately, none of the specimens bears the name “*C.symplocifolia*” or the catalogue number. Considering the name was published in Griffith’s posthumous papers, it would be unsurprising that the original material of the name had not been clearly labelled. Based on the protologue and the drawing, one of the specimens, *Griffith s.n.* (TCD0018254; Fig. 2), with flower materials (e.g. flower buds, androecia and gynoecia) and barcoded, is selected above as the lectotype of *C.symplocifolia*. Additionally, the specimen (TCD0018254; Fig. 2) can be easily distinguished from *C.caudata* Wall. that was listed on the same page of Griffith (1848) by its subsessile flower buds and glabrous filaments, whereas the latter bears pedicellate flower buds and hairy filaments.

*Camelliasymplocifolia* is treated as a heterotypic synonym of *C.kissi* Wall. by Sealy (1958: 197), Chang (1981: 35) and Ming (2000: 303). I agree with this treatment.

### ﻿3. Thea forrestii

Taxon classificationPlantaeEricalesTheaceae

Diels, Notes Roy. Bot. Gard. Edinburgh 5: 284. (1912) ≡ Camellia forrestii (Diels) Cohen-Stuart

3ACD7A58-3FBB-530A-83AD-2748A0651891

#### Lectotype.

(designated here; first-step designated by Sealy 1958: 54): China. Yunnan: South end of Tsu-hsiong-fu (Chuxiong) Valley, 7000–8000 ft alt., February 1903, *G. Forrest 314* (E00284407!; its image is available at http://data.rbge.org.uk/herb/E00284407).

#### Notes.

Diels (1912) cited a single gathering, *G. Forrest 314*, as the type of *T.forrestii* in the protologue. Cohen-Stuart (1916) transferred this species into *Camellia* without any comment on the type. However, three duplicates of *G. Forrest 314* were found at E (E00284407), K (K000380522) and P (P04511547), so they are syntypes of *T.forrestii* (Art. 40 Note 1 of the ICN). When Sealy (1958: 54) indicated “*Forrest 314* (E, K; type of *C.forrestii*)”, such citation may act as the first-step lectotypification under Art 9.17 of the ICN. It is further narrowed to a single specimen at E (E00284407) here as the second-step lectotype of *T.forrestii*.

### ﻿4. Thea lanceolata

Taxon classificationPlantaeEricalesTheaceae

(Blume) Pierre var. stenophylla Merr., Enum. Philipp. Fl. Pl. 70 (1923).

1CB2C3A9-391A-5C67-967E-49331CF8354C

#### Lectotype.

(designated here): Philippines. Luzon: Ilocos Norte Province, Bangui to Claveria, August 1918, *M. Ramos 33005* (US 00113903 [the image is available at http://n2t.net/ark:/65665/34f38884f-74b3-4f15-8e0b-db0f884e39b1]!).

#### Notes.

Merrill (1923: 70) cited a single collection, “*B.S. 33005 Ramos*” in the protologue without specifying the corresponding herbarium. Two duplicates of this gathering were found at K and US, so they are syntypes (Art. 40 Note 1 of the ICN) of T.lanceolatavar.stenophylla. The specimen at US bearing barcode 00113903 is selected above as the lectotype of this taxon because a fragment of fruit is attached on a branchlet of the sheet.

Due to a lack of materials, Sealy (1958: 145) did not give this variety a formal taxonomic treatment. Keng (1989: 68) identified it as a heterotypic synonym of *C.lanceolata* (Blume) Seem. and claimed that the latter was “understandably unlikely to be a homogeneous taxon” based on its widespread geographic distribution. I generally agree with Keng’s (1989) broad circumscription of *C.lanceolata* until more data are available (see below).

### ﻿5. Thea megacarpa

Taxon classificationPlantaeEricalesTheaceae

Elmer, Leafl. Philipp. Bot. 5: 1842. (1913)

8BEBB89C-8CD2-5E16-A406-7DD534C1799D

#### Lectotype.

(designated here): Philippines. Palawan: Puerto Princesa (Mt. Pulgar), March 1911, *Elmer 12822* (E00504323!; its image is available at http://data.rbge.org.uk/herb/E00504323).

#### Notes.

A single collection, *Elmer 12822*, was cited in the protologue (Elmer 1913: 1843) without indicating where the specimens were conserved. Ten duplicates of *Elmer 12822* housed at various Herbaria A (00025101), BM, E (E00504323), G (G00354856), K, MO (705490), NY (00385756), P (P04511437), U (U 0226169) and US (00113904) were found, so they are the syntypes of *T.megacarpa* (Art. 40 Note 1 of the ICN). The citation of Ming (2000: 228), “Type:... *A.D.E. Elmer 12822* (K, E, BM, P)”, did not validate the lectotypification because the single herbarium in which the type was deposited was not specified (Art. 9.22 of the ICN). The specimen at E (E00504323), which bears immature fruit and seeds, is selected as lectotype.

Cohen-Stuart (1916: 68) transferred *Theamegacarpa* into *Camellia*. Sealy (1958: 142) treated it as a heterotypic synonym of *C.lanceolata*. Chang and Ren (1991: 68) thought that *Elmer 12822* “much differed from” *C.lanceolata* because the former bore “free filaments and [a] thicker pericarp”, whereas the latter bore “totally united filaments” and a “thinner pericarp”. However, flowers, including filaments, were absent in all the specimens of *Elmer 12822* examined above and there was no description of flower parts in the protologue (Elmer 1913: 1842–1843). Later, Ming (2000: 228) recognised the plant as a subspecies of *C.furfuracea* (Merr.) Cohen-Stuart.

However, as a native and the single representative of *Camellia* in the Malay Archipelago, *C.lanceolata* holds a specific phylogenetic position (Zhao et al. 2022). The plants under the broad circumscription of this taxon show a continuous variation in the size and shape of the morphological characters. For instance, the length of the leaf blade can vary from 2 cm (e.g. *Beaman 8977* at K) to 13 cm (e.g. the lectotype of *T.megacarpa*, *Elmer 12822* at E), but the elements of flower and fruit, such as the filament tube, the hairy ovary and the furfuraceous surface of the pericarp, are generally similar amongst them. Since there is no clear correlation between morphological variation and geographic distribution and molecular phylogenetic analysis of the plants is absent, I provisionally agree with the broad definition of *C.lanceolata* and place T.lanceolatavar.stenophylla and *T.megacarpa* in its synonymy.

### ﻿6. Thea yunnanensis

Taxon classificationPlantaeEricalesTheaceae

Pit. ex Diels, Notes Roy. Bot. Gard. Edinburgh 5: 284. (1912) ≡ Camellia yunnanensis (Pit. ex Diels) Cohen-Stuart

2B95191D-0BEB-547C-9966-B435E31BD014

#### Lectotype.

(designated here; first-step designated by Sealy 1958: 163): China. Yunnan: Ta long tan, 10 October 1889, *Delavay s.n.* (P01903507!; its image is available at https://science.mnhn.fr/institution/mnhn/collection/p/item/p01903507).

#### Notes.

Two gatherings, *Delavay s.n.* and *G. Forrest 430*, were cited in the protologue (Diels 1912: 284), so they are syntypes of *T.yunnanensis* (Art. 9.6 of the ICN). When Sealy (1958: 163) cited “*Delavay 15 Oct. 1889* (P, type)”, his lectotypification must be accepted, but may be considered as the first-step (Art. 9.17 of the ICN) because three specimens of Delavay’s collection matching the description of *T.yunnanensis* were found at P (P01903507, P01903508 and a single specimen consisting of two sheets: P06614716 [1/2] & P06614717 [2/2]; Art. 8.3 of the ICN, also see Ex. 7). However, the three specimens of *Delavay s.n.* were all collected on 10 October 1889 and two of them, P01903507 and P01903508, bear Pitard’s handwriting “*Thea yunnanensis* sp. n.”, so the date of collection recorded in the protologue, “15 October 1889”, is probably incorrect and the correct date is 10 October 1889 which was recorded on the labels. Specimen with barcode P01903507 bears a drawing of a dissected flower and is designated above as the second-step lectotype of *T.yunnanensis* (Art. 9.17 of the ICN).

## Supplementary Material

XML Treatment for Camellia reticulata Lindl. f. simplex

XML Treatment for Camellia symplocifolia

XML Treatment for Thea forrestii

XML Treatment for Thea lanceolata

XML Treatment for Thea megacarpa

XML Treatment for yunnanensis
